# Cardiovascular Remodeling Post-Ischemia: Herbs, Diet, and Drug Interventions

**DOI:** 10.3390/biomedicines11061697

**Published:** 2023-06-13

**Authors:** Ayodeji A. Olabiyi, Lisandra E. de Castro Brás

**Affiliations:** Department of Physiology, Brody School of Medicine, East Carolina University, Greenville, NC 27858, USA; decastrobrasl14@ecu.edu

**Keywords:** cardiovascular disease, diet, herbs, pharmaceutical drugs, remodeling

## Abstract

Cardiovascular disease (CVD) is a serious health burden with increasing prevalence, and CVD continues to be the principal global source of illness and mortality. For several disorders, including CVD, the use of dietary and medicinal herbs instead of pharmaceutical drugs continues to be an alternate therapy strategy. Despite the prevalent use of synthetic pharmaceutical medications, there is currently an unprecedented push for the use of diet and herbal preparations in contemporary medical systems. This urge is fueled by a number of factors, the two most important being the common perception that they are safe and more cost-effective than modern pharmaceutical medicines. However, there is a lack of research focused on novel treatment targets that combine all these strategies—pharmaceuticals, diet, and herbs. In this review, we looked at the reported effects of pharmaceutical drugs and diet, as well as medicinal herbs, and propose a combination of these approaches to target independent pathways that could synergistically be efficacious in treating cardiovascular disease.

## 1. Introduction

According to the World Health Organization, each year 17.9 million people worldwide die from cardiovascular disease (CVD), accounting for 31% of all mortality [1]. The American Heart Association reports that roughly half of all Americans have a type of CVD [2]. Of these, heart attacks (i.e., myocardial infarction, MI) and strokes account for 80% of CVD fatalities [2]. An MI occurs when an artery to the heart is clogged, and the heart does not receive enough blood or oxygen for a significant amount of time leading to ischemia/infarction of the tissue downstream of the obstruction. Upon infarction, if reperfusion does not occur in a timely manner, cardiac cells will become ischemic and metabolically compromised.

Cardiac cells (including cardiomyocytes), capillaries, and extracellular matrix (ECM), which is made up of various collagen fiber types and ensures structural integrity, are the three crucial components of heart muscle that enable effective contraction and pumping of the blood to the whole body. When cells die due to ischemia, a cascade of events is triggered to clear and replace the damaged cells and tissues. These events are dynamic and are broadly divided into three stages: inflammation/necrosis to remove dead cells/tissue; proliferation/fibrosis to replace cells, ECM, and set up tissue vascularization; and long-term remodeling/maturation to regenerate the tissue and/or mature the scar and provide tissue integrity and function [3]. Different risk cues, either exogenous or endogenous, may start the inflammatory response. Microorganism invasion is considered exogenous, whereas tissue injury is considered endogenous. Pathogen-associated molecular patterns (PAMPs) and damage-associated molecular patterns (DAMPs) are the names given to the external and endogenous signaling molecules that are released, respectively, by pathogens or necrotic cells [4]. Toll-like receptors (TLRs), nucleotide-binding oligomerization domain (NOD)-like receptors (NLRs), C-type lectins, and receptors for advanced glycation end-products (RAGE) are just a few of the pattern recognition receptors (PRRs) that may detect PAMPs and DAMPs. A described genus of specific pro-resolving mediators (SPMs) including lipoxins, resolvins, protectins, and maresins is activated during the resolution phase of inflammation [5]. These mediators have strong, direct anti-inflammatory and pro-resolution properties that limit the recruitment of inflammatory leukocytes while encouraging macrophage clearance of apoptotic neutrophils. By controlling ventricular size, shape, and function, left ventricular (LV) remodeling defines the heart’s (mal)adaptation to injury and/or mechanical, neurohormonal, and hereditary alterations. While the increased microcirculatory blood supply provided during the proliferation phase will support any live cardiomyocytes, continual LV remodeling (due to uncontrolled inflammation) leads to cardiomyocyte loss, expansion of the ischemic area, persistent collagenase activity, and degradation of high-tensile collagens; this in turn leads to LV dilation, with wall thinning and fibrosis [6]. This “adverse” or “pathological” remodeling following MI is maladaptive, carries a disproportionate risk of heart failure (HF), and dramatically lowers survival [7]. Over the last twenty years, the prevalence of HF has increased [8]. Despite immediate revascularization and subsequent treatment options that significantly lower the mortality of acute MI, ischemic heart disease remains the main cause of HF [8,9]. Comorbidities foretell the severity and progression of HF [10,11]; in fact, more than 50% of HF patients have more than seven comorbidities, with age and sex being the two main predictors of HF [12]. Men have a larger heart mass and a higher incidence of subclinical coronary artery disease than women [13,14]. Women show less severe incidence than men in terms of atherosclerosis, left ventricular hypertrophy, and cardiomyocyte apoptosis [15,16]. Similarly, mortality post-MI correlates with advancing age, regardless of infarct size [17], which may be related to a higher prevalence of ventricular hypertrophy but also to a decreased immunological response, scarring, and autophagy [18].

Medications that have been effective in treating HF include neurohormonal antagonists (beta blockers, angiotensin receptor blockers, and aldosterone antagonists), which can prevent or reverse remodeling and have been shown to lower mortality and morbidity [19]. The use of medications has been practiced over the years for the prevention of HF. However, some of these medications produce adverse effects and due to high cost are not easily available to every patient. Over the past three decades, there has been a significant rise in the use of herbal supplements to prevent, avoid, and/or treat different conditions, including CVD; this rise results from a perceived safety (natural, non-toxic sources), efficiency with few or no adverse effects (unlike synthetic/chemical medications), and low cost [20]. The US Dietary Supplement Health and Education Act (DSHEA) of 1994 categorizes herbal products as dietary supplements. In addition to herbal supplements, epidemiological research showed that consuming fruits, vegetables, olive oil, wine, legumes, and whole grains, as found in the Mediterranean diet, has a cardioprotective effect [21]. Foods high in polyphenols, such as berries and olive oil, have been demonstrated to have cardioprotective benefits that enhance endothelial function and plasma lipid profiles while preventing aberrant platelet aggregation and decreasing inflammation [22]. Thus, diet and dietary supplements have important roles in maintaining homeostasis and reducing CVD and warrant further investigation. Preload, afterload, and molecular mechanisms of remodeling have been targeted using synthetic drug therapy to date; can this also be achieved through diet and natural supplements? This review focuses on the use of prescription drugs, herbal products, and diet modification on cardiovascular remodeling.

## 2. Cardiovascular Health and Drugs Treatment

Over the past two centuries, the pathways of discovery, innovation, and therapeutic improvement in cardiovascular science and medicine have been really astounding. Nabel and Braunwald [23] discussed the remarkable decrease in cardiovascular disease deaths due to scientific advancements from 1950, when it was over 450 deaths per 100,000 of the population, to approximately 100 deaths per 100,000 by 2010. Even though many clinical advancements have contributed to this impressive decline, they emphasized the significance of β-blocking therapy and 3-hydroxy-3-methyl-glutaryl coenzyme A (HMG Co-A) reductase inhibition, as well as the introduction of novel antihypertensive agents in the context of the national high blood pressure education program. Nevertheless, notwithstanding these medical advances, concurrent disorders such as diabetes mellitus, obesity, and congestive HF contribute to the consistently high prevalence of CVD. Additionally, because of the remarkable decline in MI mortality rates, affected individuals live longer but nearly 40% develop HF, which has a worse prognosis than acute MI. Despite prior advancements in pharmacological therapy, such as oral diuretics and medication treatment for hypertension, progress is still being made in the management of cardiovascular disorders such as HF and cardiac arrhythmias.

HF patients with systolic LV dysfunction treated with angiotensin-converting enzyme (ACE) inhibitors present reduced mortality and morbidity [24]. ACE inhibitors act as vasodilators and their effectiveness in reducing remodeling is supported by both experimental and clinical studies, particularly in the post-infarction setting [20,21,22,25,26,27]. However, with ACE inhibitors, reverse remodeling has infrequently been observed in sizable patient cohorts. One of the aspects of this therapy that may limit its ability to provide long-term benefit is protection without reversion of remodeling. In clinical studies with ACE inhibitors, the survival curves of patients assigned to either the active treatment or the placebo groups typically show an early divergence of the curves followed by a parallel trend through time [28,29]. This shows that these medications more often delay the onset of the disease than stop it [30].

Angiotensin receptor blockers (ARBs) are also used in HF treatment, instead of or in combination with ACE inhibitors. ARBs selectively reduce the activity of type 2 receptors (AT2R), which mediate vasodilation and may prevent myocardial fibrosis, without affecting type 1 receptors (AT1R), which primarily mediate cardiac hypertrophy, aldosterone production, fibrosis, and vasoconstriction [31].

Matrix metalloproteinase (MMP) activation and maladaptive LV remodeling have a clear cause-and-effect relationship, as shown by experimental research [32,33,34]. Thus, MMPs have long been recognized as an attractive target to decrease or prevent maladaptive cardiac remodeling. MMP inhibitors have been suggested as a treatment for several illnesses in order to lessen matrix deterioration, blunt inflammation, and enhance tissue regeneration. However, difficulties in producing MMP specific inhibitors and the many functions of MMPS (both beneficial and detrimental) have led to little progress in clinical trials and still need further investigation [35,36,37].

## 3. Cardiovascular Health and Diet

According to data from the Centers for Disease Control (CDC) and Prevention, some of the most common health problems in the United States are chronic diseases such as cancer, diabetes, heart disease, and stroke. Many of these chronic illnesses can be prevented, though, as they are linked to unhealthy eating and lifestyle choices. Dietary intervention allows for a better blending of various diets and nutrients. Healthy eating practices therefore encourage a greater magnitude of favorable impacts than the potential effects of a single nutrient supplementation because of the synergistic health effects among them. Research has shown that a high intake of fiber, antioxidants, vitamins, minerals, polyphenols, monounsaturated fatty acids (MUFA), and polyunsaturated fatty acids (PUFA), low intakes of salt, saturated fats, and trans fats, and a high intake of carbohydrates with low glycemic loads are all indicators of healthy eating habits [38].

Although some cardiovascular diseases are congenital, years of investigation into the relationship between diet and heart disease concentrated on specific vitamins and minerals, types of fats, and individual nutrients such as cholesterol (and foods high in dietary cholesterol, such eggs). This has been eye-opening, but it has also led to some dead ends and perpetuated misconceptions about what a heart-healthy diet is. People eat food, not nutrition, which explains this [38]. A growing heart need nutritious grains, vegetables, and fruits, as well as a mineral balance (sodium and potassium) and body weight maintenance, as well as sufficient sleep. People who follow this eating pattern have a 31% lower risk of heart disease, a 33% lower risk of diabetes, and a 20% lower risk of stroke [39]. Neonatal malnutrition, inflammation, and growth retardation are linked to a lifelong decrease in cardiomyocyte number, systolic and diastolic dysfunction, ischemia sensitivity, and hypertension [40,41,42,43]. A significant body of scientific research supports nutrition as one of the most effective ways to avoid CVD associated mortality [44] and may even be able to reverse heart disease [45]. Even more, the management of other risk factors, such as excess weight, hypertension, diabetes, or dyslipidemia, appears to depend heavily on nutrition and exercise [45]. In this regard, foods or dietary habits that can improve CVD prevention have been identified and categorized.

### 3.1. Mediterranean Diet

The Mediterranean diet (MeDiet), known as a mainly plant-based diet, characterized by a high consumption of fruits, vegetables, and whole grains as well as fish, nuts, and legumes, unsaturated fatty acids, and low to moderate intake of alcohol (consumed with a meal) has been reported to have a great impact on the arterial wall, which could account for the association between MeDiet and low CVD prevalence [46]. Intriguingly, MeDiet appears to modulate the expression of pro-atherogenic genes like cyclooxygenase-2 (COX-2), monocyte chemoattractant protein-1 (MCP-1), and low-density lipoprotein receptor-related protein (LRP1), while decreasing plasmatic levels of molecules negatively linked to plaque stability and rupture, such as MMP-9 and interleukins (IL-10, IL-13, or IL-18) [47,48]. Furthermore, in order to reduce the risk of CVD, the American Heart Association Nutrition Committee and the European Society of Cardiology both strongly recommend daily consumption of several portions of both fruits and vegetables [49,50], hallmarks of the MeDiet. These recommendations are mostly supported by epidemiological studies and meta-analyses [49,50,51,52,53]. According to a meta-analysis [52] of 83 studies, an increase in the consumption of fruits and vegetables was significantly inversely related to C-reactive protein (CRP) and tumor necrosis factor (TNF) levels and directly related to an increase in the proliferation of T-cell populations (71 clinical trials and 12 observational studies). The main component of the MeDiet, extra virgin olive oil (EVOO), has bioactive components shown to improve endothelial dysfunction, oxidative stress, and overall inflammatory state [54]. A meta-analysis by Schwingshackl et al. [55] found that, compared to controls, daily ingestion of 1 mg and 50 mg of olive oil significantly reduced CRP in 30 randomized control studies with a total of 3106 participants. Additionally, those who consumed the most olive oil had a substantially higher flow-mediated dilatation score (0.6%, *p* < 0.002). Similar to EVOO, nuts contain monosaturated fats; numerous sizable studies have shown that nuts significantly lower CVD morbidity and death [56,57,58,59]. Hence, a MeDiet rich in nuts had been linked to better weight reduction, lower low-density lipoprotein-cholesterol (LDL-c) levels [60,61,62], lower risk of developing hypertension [61,63], and lower levels of inflammatory and oxidative mediators [64,65].

### 3.2. Paleo Diet

Another eating plan based on foods popular during the Old Stone Age is the Paleolithic diet (Paleo), which primarily consists of lean meat, fish, eggs, fruits, vegetables, roots, and nuts while avoiding grains, dairy products, processed foods, added sugars, and salts [66]. This eating plan has less salt but more protein, several micronutrients such as vitamins C and E, carotenes, and fiber [67]. It also has fewer calories from refined carbohydrates and fat [67,68,69]. According to a meta-analysis of 8 studies, a Paleo diet significantly improved circulating concentrations of total cholesterol, triglycerides, LDL-c, and CRP while also significantly lowering body weight, waist circumference, body mass index, body fat percentage, and systolic and diastolic blood pressure. It was also reported to significantly increase HDL cholesterol [70]. Another meta-analysis of four randomized control trials (RCTs) suggested short-term improvements in metabolic syndrome components following adoption of a Paleo diet, as reported by Manheimer et al. [71].

### 3.3. Keto Diet

Diets low in carbohydrates, but usually high in fats and/or proteins, have also become increasingly popular, referred to as ketogenic diets (keto diet). A keto diet restricts carbohydrate intake to less than 25 to 50 g per day, in an attempt to induce tissues to use fat or ketones as fuel during caloric restriction. As a result, the liver begins to produce ketone bodies, which are then utilized as an alternative energy source, particularly by the central nervous system [62]. From several reports, the keto diet may be linked to some improvements in various cardiovascular risk factors, such as obesity, type 2 diabetes, and HDL cholesterol levels [72,73,74,75,76,77]. On the other hand, a recent report describes keto diet causing cardiac fibrosis and inhibiting mitochondrial biogenesis [78]. According to their study, keto diet or repeated deep fasting impaired mitochondrial biogenesis, decreased cell respiration, and enhanced cardiac fibrosis and death in cardiomyocytes. As revealed by the research, increased levels of the histone deacetylase 2 (HDAC2) and inhibitor ketone body -hydroxybutyrate (-OHB), which promotes histone acetylation of the Sirt7 promoter and activates Sirt7 transcription, caused an increase in cardiac fibrosis and cardiomyocyte apoptosis [75]. Sirt7 transcription also prevents the transcription of mitochondrial ribosome-encoding genes. According to Xu and colleagues, consuming a keto diet over an extended period or accumulating -OHB may raise the chance of developing cardiovascular disease. In another population-based study, a low-carbohydrate diet, similar to keto, resulted in deleterious effects on the coronary artery [79]. In contrast, a research study in 2020 showed a keto diet was beneficial in diabetic patients by promoting cardiomyocyte survival and reducing interstitial fibrosis [79]. Although it is not clear what the reason(s) are for the discrepancies observed within these studies, it could result from differences between the animal models.

### 3.4. DASH

In 1990, dietary approaches to stop hypertension (DASH) were initiated; these approaches have received attention and several grants from the National Institute of Health (NIH) to see if specific dietary interventions were useful in treating hypertension. Some of the individuals enrolled showed a 6 to 11 mm Hg reduction in systolic blood pressure from the food intervention alone [80]. Both hypertensive and normotensive individuals experienced this effect, and as a result, DASH has occasionally been recommended as the first-line pharmacologic therapy in conjunction with lifestyle modifications. DASH encourages the eating of fresh produce, lean meat, low-fat dairy products, and foods rich in micronutrients. Additionally, it encourages limiting daily sodium intake to 1500 mg. DASH places a strong emphasis on eating fresh, minimally processed foods. The DASH diet is very similar to other dietary styles that are supported for cardiovascular health. The DASH diet is a synthesis of the old and new [81,82,83].

## 4. Cardiovascular Health and Herbal Medicine

Traditional herbal remedies have been used for decades and are typically regarded as safer than synthetic pharmaceuticals [84,85,86]. Approaches influenced by traditional medicine are still crucial, particularly for the management of chronic illnesses and to speed up the development of natural product drugs [87,88]. When administered with contemporary synthetic treatments, combinations of herbal medications or phytochemical active ingredients have been demonstrated to be useful in treating several disorders [89]. While the market for dietary botanical supplements is expanding, it is strongly recommended that more thorough clinical and scientific research be conducted on herbal and traditional medicines to increase their acceptance and visibility. Moreover, it is necessary to increase control on the quality of the materials used for formulation of the herbal/dietary supplements to ensure efficacy and safety.

In plants, certain polyphenols are present with adequate bioavailability singly or in combination with others (acting additively or synergistically). According to laboratory data gathered over the years, medicinal herbs can affect several CVD risks factors and, therefore, may have therapeutic utility in treating CVDs (Table 1). To effectively use herbs in CVD therapy, there have been numerous initiatives to shift studies on medicinal herbs from the bench to the bedside [90,91]. Natural products’ biological activity and structural variety outperform any existing synthetic drug screening library, which is another factor leading to a return of interest in them [90].

The herbs *ginseng*, *ginkgo biloba*, *ganoderma lucidum*, and *gynostemma pentaphyllum* have been reported to prevent CVDs [92,93]. These herbs are becoming more and more well-known due to their presence in commercial commodities in numerous markets around the world and their established therapeutic potential in a variety of settings, including cardiovascular conditions [92]. These organic compounds profoundly alter important cellular, molecular, and metabolic processes that regulate the etiology and pathogenesis of CVDs [94]. Recent research shows that these herbal remedies have strong therapeutic effects and can improve pathological diseases linked to CVDs [92,93]. Clear clinical therapeutic advantages have not, however, been established. Rafacho et al. [95] demonstrated rosemary supplementation (*Rosmarinus oficinallis* L.) to cause reduced myocardial damage and blood pressure in hypertensive rats fed a high-fructose diet and protected the heart against cardiac dysfunction and fibrosis after MI in rats. From their findings, it was concluded that the mechanism could involve improved energy metabolism and reduced oxidative stress. In general, herbs could prevent or treat more than one disease since they have several therapeutic properties. As an illustration, the herb *Crocus sativus* L. was discovered to contain therapeutic properties that may be used to treat five different types of heart illness, including hypertension, heart attacks, blood fat reduction, antioxidants, and cardiac tonics [96,97,98]. This herb’s role in preventing cardiovascular disorders was reportedly attributed to its anti-inflammatory and antioxidant properties [84,99].

In another study, due to strong antioxidant and free radical-scavenging activity, *Citrus medica* L. has also been reported to have cardioprotective potential [100]. In addition, a *Crataegus* species was observed to be a safe, nontoxic therapy option for ischemic heart disease and cardiovascular disease [101]. For example, the fruit or, alternatively, the leaves and flowers of *Crataegus monogyna*, which are high in polyphenols, are used medicinally. Direct scavenging of reactive oxygen species (ROS), increased catalase and superoxide dismutase activities, antioxidant activity, and downregulation of the caspase 3 gene are some of the known mechanisms of action of the *Crataegus* species [101]. Hawthorns are frequently utilized in a holistic approach to treat circulatory problems and congestive heart failure [102]. Moreover, stage 1 hypertension individuals who consume *Elettaria cardamom* have significantly lower blood pressure, increased fibrinolysis, and improved antioxidant status [103]. A recent study investigated the ability of cardamom oil to reestablish lipid homeostasis in the presence of hypercholesterolemia [104]. This study reduced atherogenicity index with dietary intervention with cardamom powder and oil, demonstrating the cardioprotective benefit of cardamom [104]. Researchers have also discovered two major methods through which the bark of *Terminalia arjuna* demonstrates cardioprotective advantages against induced cardiotoxicity which includes increased coronary artery flow and protection of the myocardium from ischemic damage [105].

**Table 1:** List of herbal remedies traditionally used for the treatment of different forms of CVDs.

Herbs	Forms of CVDs	Reference
*Ginseng*	Oxidative stress, hypertension, cardiac disease, hyperlipidemia and ion regulation	[93,106]
*Ginkgo biloba*	Cardiac activity, vasorelaxant and vasoconstriction activity, hypertension	[93]
*Ganoderma lucidum*	Atherosclerosis, hyperlipidemia	[107]
*Gnostemma pentaphyllum*	Hyperlipidemia	[92]
*Rosemary oficinalis* L.	Cardiac dysfunction and fibrosis	[95]
*Cocus sativus* L.	Systolic hypertension, oxidative stress, inflammation	[86,92]
*Citrus medica* L.	Ischemia heart disease	[100]
*Crataegus monogyna*	Congestive heart failure	[101]
*Elettaria cardamom*	Hypercholesterolemia	[104]
*Terminalia arjuna*	Cardiotoxicity	[105]
*Punica granatum* L.	Blood pressure, inflammation	[108,109]
*Apple* (*Malus pumila*)	Blood lipid levels	[110,111]
*Watermelon* (*Citrullus lanatus*)	Heart attacks, ischemic strokes, atherosclerosis	[112,113,114]
*Berries*	Myocardial infarction, oxidative stress, inflammation, platelet aggregation	[115,116,117]
*Grapes* (*Vitis vinifera* L.)	Cardiac fibrosis, hyperlipidemia	[118,119]
*Garlic* (*Allium satinum* L.)	Hypertension, hypercholesterolemia	[120]
*Cinnamon* (*Cinnamomum verum*)	Oxidative stress, inflammation, artherosclerosis	[121]

## 5. Polyphenols and Cardiovascular Health

**Figure 1:**
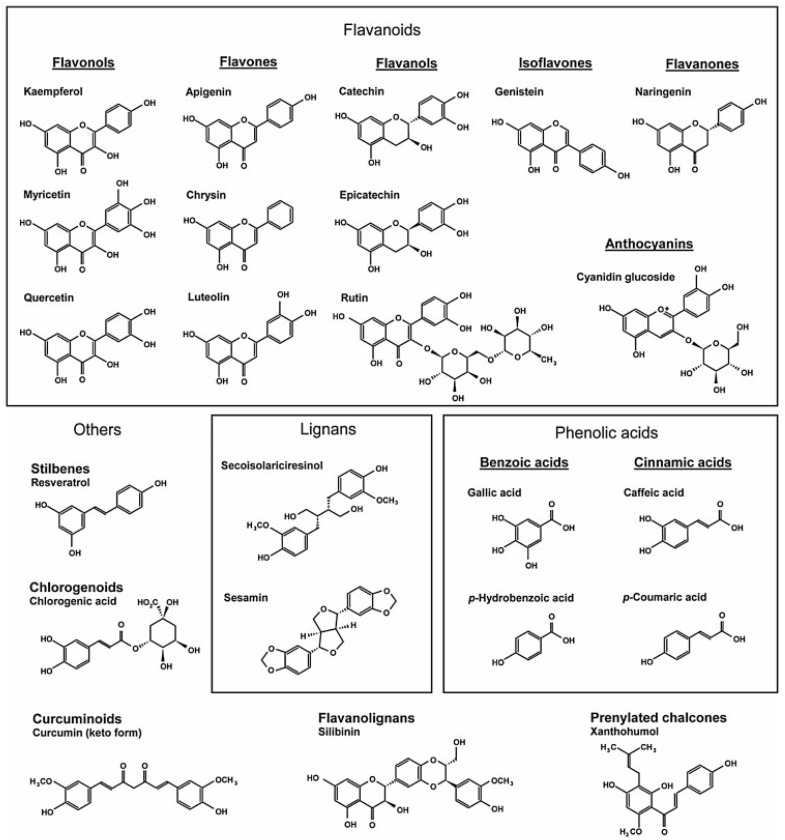
Chemical compositions of the various polyphenol classes. The amount of phenol rings a polyphenol contains and the structural components that hold these rings together are used to categorize polyphenols. They can be generally divided into four classes: stilbenes, lignans, phenolic acids, and flavonoids. Hydroxyl benzoic and hydroxyl cinnamic acids are two more subgroups of phenolic acids. Phenolic acids, which are present in all plant material but are more prevalent in fruits with an acidic flavor, make up around one-third of the polyphenolic substances in our diet. Some common phenolic acids include ferulic acid, gallic acid, and caffeic acid. The most prevalent polyphenols in the human diet are flavonoids, which have a basic structure that consists of two aromatic rings connected by three carbon atoms to create an oxygenated heterocycle. Biogenetically, one ring typically develops from a resorcinol molecule, while the other ring develops from the shikimate route. Two phenyl moieties are joined by a two-carbon methylene bridge in stilbenes. The majority of stilbenes in plants function as phytoalexins, which are substances that are only produced in reaction to infection or damage. Resveratrol has been the stilbene that has been investigated the most. The 2,3-dibenzylbutane structure of lignans, which are diphenolic substances, is created when two cinnamic acid residues dimerize.

Plants and plant products have been documented to have over 8000 known polyphenols [122]. Human health and wellness have been shown to directly be affected by a polyphenol-rich diet. According to Quideau et al. [123], polyphenols are substances with several phenolic units and no nitrogen-based functionalities that are generated from the polyketide and/or shikimate/phenyl propanoid pathways. After being metabolized, most polyphenols are glycosylated and either can bind to other phenols or conjugate with glucuronic acid, galacturonic acid, or glutathione [124]. Polyphenols are categorized into four main types: phenolic acids, stilbenes, lignans, and flavonoids (Figure 1).

### 5.1. Phenolic Acids

Phenolic acids include caffeic acid, which can be found in almost all fruits; chlorogenic acid, found in strawberries, pineapple, and other foods; and *p*-coumaric acid, present in cereal grains. The two families of phenolic acids—derivatives of benzoic acid and derivatives of cinnamic acid—are widely present in foods. Except for some red fruits, black radishes, and onions, which can have concentrations of many tens of milligrams per kilogram fresh weight of hydroxybenzoic acid, the hydroxybenzoic acid content of food plants are often modest [125].

### 5.2. Stilbenes

Stilbenes are phenolic substances with two phenyl groups joined by a bridge made of two carbon atoms in the methylene ring. Most stilbenes in plants function as phytoalexins, which are substances that are often only produced as a result of infection or injury. Resveratrol is one of the most thoroughly investigated stilbenes. Red wines, red grape juice, and peanuts all contain stilbenes [126,127].

### 5.3. Lignans

A wide class of low molecular weight polyphenols called lignans is present in many types of plants, especially seeds, whole grains, and vegetables. The word “wood” in Latin is the source of the name. Phytoestrogens’ predecessors are lignans [128]. They might act as antifeedants to protect seeds and plants from herbivores [129]. Secoisolariciresinol from flaxseed [130] and sesamin from sesame seed [131] are two major examples of lignans.

### 5.4. Flavonoids

Fruits, vegetables, legumes, red wine, and green tea all contain flavonoids. They are further divided into chalcones, anthocyanins, proanthocyanidins, flavanols, flavonols, flavones, isoflavones, and flavanones [97]. Green and black tea include flavanols, such as epigallocatechin gallate [132]. Onions, broccoli, and blueberries all contain flavonols, such as kaempferol and quercetin [133]. In strongly colored fruit, anthocyanins can be discovered (cyanin glucoside is one of the examples) as reported by Ly et al. [124]. Parsley and celery both contain flavones, such as apigenin, chrysin, and luteolin [124]. Soya and its processed derivatives contain isoflavones, such as daidzein and genistein [134]. Grapefruit contains flavanones, such as naringenin [124]. Chalcones are primarily plentiful in hops used in beer making. They are also plentiful in numerous plants and spices, as well as in several vegetables and fruits, such as shallots, tomatoes, potatoes, and bean sprouts (as well as licorice and cardamom). The primary prenylated chalcone is xanthohumol, which is mostly present in hops and hence in beer [135]. Condensed flavan-3-ols known as proanthocyanidins are present in numerous plants, including apples, grape seeds, grape skin, and cocoa beans (the main source) [136]. Another group of polyphenols is the curcuminoids which includes turmeric or the compound curcumin.

Generally, the most prevalent polyphenols among these several types are phenolic acids and flavonoids [137]. Various protective properties of natural polyphenols against cardiovascular illnesses have been reported [59,88,138,139]. It has been noted that polyphenols belonging to various subclasses can reduce heart fibrosis and dysfunction after a cardiac injury. Quercetin with its glycoside derivative, rutin, or solitary quercetin reduced cardiac dysfunction and myocardial damage brought on by isoproterenol, prevented cardiac fibrosis, and inhibited the synthesis of connective tissue growth factor (CTGF), transforming growth factor-β (TGF-β1), and ECM components [140]. Panchal et al. [141] reported that by blocking the NF-κB signaling pathway and promoting nuclear factor erythroid 2-related factor 2 (Nrf-2) and its downstream components, heart remodeling was prevented in an obese rat model fed a Western diet supplemented with quercetin.

The most common and powerful catechin in green tea is epigallocatechin-3-gallate (EGCG) [142]. In rats, EGCG has been reported to reduce heart hypertrophy and the proliferation of cardiac fibroblasts (CFs) [143]. Other findings revealed EGCG inhibited oxidative stress, which slowed heart hypertrophy both in vivo and in vitro [144]. In the meantime, it reduced the activation of rat CFs induced by AngII through the involvement of β -arrestin1 [145]. By blocking the NF-κB signaling pathway during hypertrophic stimulation, Cai et al. [146] discovered that EGCG might inhibit the expression of fibronectin and collagen formation in rat CFs brought on by AngII. It also significantly improved excessive CTGF expression and cardiac fibrosis [146]. An appropriate dose is necessary for EGCG to play its protective effect in reducing heart fibrosis, though. In contrast, a high dose of EGCG causes the creation of cardiac collagen and worsens cardiac fibrosis in mice [147].

NADPH oxidases (NOXs), a class of enzymes linked to the production of ROS, were found to contribute to the advancement of myocardial fibrosis and HF [148]. The myocardium expresses all forms of NOX, but NOX2 and NOX4 are predominant. In disease, the human heart’s end-stage failure exhibits enhanced NOX expression, and cardiac hypertrophy is evidently a significant source of elevated cardiac ROS. According to Wang et al. [149], luteolin inhibits CFs proliferation by reducing oxidative stress in vitro. The mechanism underlying this effect is dependent on the regulation of NOX2 and NOX4 in cardiac hypertrophy, which reduces the production of c-Jun N-terminal kinase (JNK) and TGF-1 β and lowers cardiac fibrosis [150]. Additionally, the anthocyanins found in grape skins, such as malvidin-3-glucoside, delphinidin-3-glucoside (Dp3G), cyanidin-3-glucoside (Cy3G), petunidin-3-glucoside (Pg3G), and peonidin-3-glucoside, guard against problems from ischemia/reperfusion and diabetic mellitus [151]. Dp3G and Cy3G, but not Pg3G, were able to restore complex I of the mitochondrial respiratory chain to its original condition and increase ischemia-depleted ATP levels via promoting oxidative phosphorylation [152]. Because of its high potential to decrease cytosolic cytochrome c, Cy3G, but not Pg3G, protects the rat heart against ischemia/reperfusion-induced apoptosis and necrosis [153]. Administration of Cy3G improves cardiac dysfunction and cardiac inflammation in STZ-induced diabetic cardiomyopathy by activating MMP-9 and lowering the level of tissue inhibitor of MMP-9 (TIMP-1) found in diabetic rat hearts [154].

Curcumin, a natural inhibitor of p300-specific histone acetyltransferase, has been demonstrated to prevent HF [155]. Curcumin alone or in combination with enalapril reduces severe perivascular fibrosis in rats after MI and enhances left ventricular systolic function via reducing nuclear expression of p300 [155,156]. According to a different study, curcumin prevents unfavorable cardiac repair and lessens the fibrotic response to ischemia and reperfusion by slowing the breakdown of the extracellular matrix and preventing the synthesis of collagens through the TGF-β/Smad pathway [157]. Curcumin dramatically reduced collagen buildup in vivo, as well as CF proliferation, migration, and MMP production, according to another line of study [158]. According to Zeng et al. [159], curcumin’s ability to up-regulate Nrf-2 expression and suppress NF-κB activation was directly related to its protective role. Resveratrol administration has been shown to prevent and/or slow down the progression of cardiac remodeling in an animal model of HF [160].

Due to their biological actions as an antioxidant, an anti-inflammatory, and an anticancer agent, polyphenols are commonly present in many foods and have received significant attention. Polyphenols are chemicals with a benzene ring structure with two or more phenolic hydroxyl groups and, depending on their structural properties, they can be divided into flavonoids and phenolic acids. Several positive impacts of polyphenols on health have been documented [161,162,163,164,165,166]. They are endowed with special functional capabilities to exercise their good effects on human health and are added to meals owing to their remarkable biological activities, such as antioxidant and antibacterial effects, as well as their natural availability and biocompatibility. In the prevention of numerous chronic diseases such as diabetes, hypertension, and cancer, the potential significance of functional foods containing polyphenolic chemicals is of importance.

Flavonoids are divided into six main subclasses based on the variety in the type of heterocycle involved: flavonols, flavanones, flavanols, flavones, anthocyanins, and isoflavones. Individual variances within each group are caused by differences in the quantity, arrangement, alkylation, and/or glycosylation levels of the hydroxyl groups. The C ring of flavonols (such as quercetin and kaempferol) has a 3-hydroxy pyran-4-one group. The C ring of flavanones (such as naringenin and taxifolin) contains an unsaturated carbon-carbon bond. The 4-one structure in the C ring and the 3-hydroxyl group are absent from flavanols (such as the catechins). There is no hydroxyl group in the 3-position on the C ring of flavones (such as luteolin). Isoflavones, such as genistein, have the B ring connected to the C ring in the 3-position rather than the 2-position as is the case with other flavonoids. Anthocyanins, such as cyanidin, are characterized by the presence of an oxonium ion on the C ring and are strongly colored as a result.

## 6. Food-Drug and Herb-Drug Interactions

Drug release, absorption, distribution, metabolism, and/or elimination can be significantly impacted by the concurrent consumption of food or herbs and medications, which can also have a negative/positive impact on the efficacy and safety of pharmacotherapy. Food and herb consumption alters the human gastrointestinal (GI) tract’s physiological environment in a number of ways that can influence a drug’s release, absorption, distribution, metabolism, and/or elimination. These interactions between food, herbs, and drugs are general, thus they will apply to any oral formulation [167]. Their applicability, however, depends on the drug’s formulation and physical characteristics. It is possible to lessen negative drug reactions and iatrogenic disorders by increasing understanding of interindividual variation in drug breakdown capability and results regarding the influence of drugs, nutrition, and herbal items. Medical care needs to be increasingly individually adjusted to each patient, and when diet and herbal treatments are administered in conjunction with medications, this can boost therapeutic efficacy and reduce drug toxicity. Designing a patient’s ideal personal regimen can be aided by understanding how dietary components might increase or decrease pharmacological efficacy. Understanding all the variables can aid in creating individualized medicine, nutrition, and herbal remedy plans for patients, preventing negative side effects, and fostering healing and health [168]. The synergistic impact of some herbs with some drugs to lessen xenobiotic inputs may be intriguing in that situation. Herbs/foods that boost a prescription drug’s effectiveness may allow for lower drug doses when taken concurrently—a potentially advantageous interaction.

## 7. Conclusions

**Figure 2:**
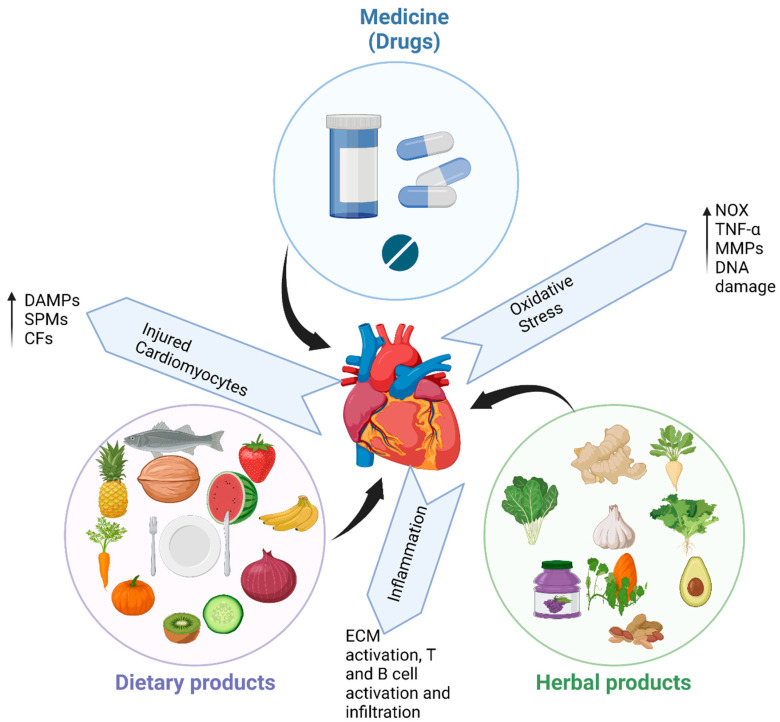
Diagrammatic representation of the influence of synthetic pharmaceutical medications, dietary and herbal products in CVD management, with emphasis in common pathways that are targeted by each intervention. DAMPs = damage-associated molecular patterns, SPMs = specific pro-resolving mediators, CFs = cardiac fibroblasts, NOX = NADPH oxidases, TNF-α = tumor necrosis factor, MMPs = metalloproteinases, DNA = deoxyribonucleic acid, ECM = extracellular matrix. Figure created with Biorender.com.

Numerous epidemiological studies have found a link between consuming natural polyphenols and a lower risk of developing chronic diseases. The risk of developing major cardiovascular illnesses, MI, and cardiovascular mortality is inversely correlated with higher fruit and vegetable consumption [169]. Synthetic medications, herbal medicine and diets have been utilized in the management of CVDs for decades. It is important to note that though many reports establish that the molecular mechanisms of cardiac remodeling have been improved using these therapies individually and not as combinations medication of HF, little research has investigated new therapeutic targets that incorporate all three tactics. While their possible interactions should be considered, a combination of treatments that target independent pathways could proof very efficacious in the treatment of CVD (Figure 2).

## Data Availability

Not applicable.
